# A data mining approach for identifying novel target specific small molecules

**DOI:** 10.1186/1755-8166-7-S1-P84

**Published:** 2014-01-21

**Authors:** Varun Khanna, Alok Dhawan

**Affiliations:** 1 Institute of Life Sciences, Ahmedabad University, Opp. University Bus Stand, Navrangpura, Ahmedabad - 380009, Gujarat, India

## Background

There has been a paradigm shift in drug discovery from being a single-target approach to a multi-target comparative analysis. This has shifted the emphasis from designing lead candidates with desirable pharmacokinetic properties against individual targets to synthesizing small molecules active at family and subfamily levels. Therefore, in the present study, protein targets and small molecule ligands available in PubChem BioAssay and DrugBank databases were comparatively analyzed to identify novel target specific small molecules.

## Materials and methods

The data obtained from public databases was mined using shell scripts and R-statistical computing software (2.15.3) installed under Linux environment.

## Results

Our data from small molecule analysis showed that 210 FDA approved drugs bind to a single protein target whereas 157 bind to two targets, 82 bind to three targets and rest bind to four or more targets. This shows that out of 1541 FDA approved drugs only 14% are specific binders whereas majority of the drugs are promiscuous binders. Similarly in PubChem BioAssay dataset 20% of the compounds bind to a single target whereas 11% compounds bind to two targets while the rest of the compounds bind to three or more targets. Further, our results from the comparison of target datasets showed that there are 2097 and 1271 unique domains in DrugBank and PubChem BioAssay target datasets respectively. There are 1190 domains common to protein targets in DrugBank and PubChem BioAssay datasets. Further, we note that of the top 10 domains 8 domains are shared between both the datasets.

## Conclusions

The above observations have implications in drug design approaches where the goal is to find target specific small molecules. Our analysis shows that promiscuity plays a major role in drug discovery therefore, should not be overlooked while designing novel drugs. From domain analysis of target proteins we conclude that protein target space is fairly narrow and the majority of the drug design efforts are concentrated only on few target classes containing limited domains.

